# The Role of Iron-Induced Fibrin in the Pathogenesis of Alzheimer’s Disease and the Protective Role of Magnesium

**DOI:** 10.3389/fnhum.2013.00735

**Published:** 2013-10-29

**Authors:** Boguslaw Lipinski, Etheresia Pretorius

**Affiliations:** ^1^Joslin Diabetes Center, Harvard Medical School, Boston, MA, USA; ^2^Department of Physiology, Faculty of Health Sciences, University of Pretoria, Arcadia, South Africa

**Keywords:** Alzheimer’s disease, iron, fibrin, red blood cells, magnesium

## Abstract

Amyloid hypothesis of Alzheimer’s disease (AD) has recently been challenged by the increasing evidence for the role of vascular and hemostatic components that impair oxygen delivery to the brain. One such component is fibrin clots, which, when they become resistant to thrombolysis, can cause chronic inflammation. It is not known, however, why some cerebral thrombi are resistant to the fibrinolytic degradation, whereas fibrin clots formed at the site of vessel wall injuries are completely, although gradually, removed to ensure proper wound healing. This phenomenon can now be explained in terms of the iron-induced free radicals that generate fibrin-like polymers remarkably resistant to the proteolytic degradation. It should be noted that similar insoluble deposits are present in AD brains in the form of aggregates with Abeta peptides that are resistant to fibrinolytic degradation. In addition, iron-induced fibrin fibers can irreversibly trap red blood cells (RBCs) and in this way obstruct oxygen delivery to the brain and induce chronic hypoxia that may contribute to AD. The RBC-fibrin aggregates can be disaggregated by magnesium ions and can also be prevented by certain polyphenols that are known to have beneficial effects in AD. In conclusion, we argue that AD can be prevented by: (1) limiting the dietary supply of trivalent iron contained in red and processed meat; (2) increasing the intake of chlorophyll-derived magnesium; and (3) consumption of foods rich in polyphenolic substances and certain aliphatic and aromatic unsaturated compounds. These dietary components are present in the Mediterranean diet known to be associated with the lower incidence of AD and other degenerative diseases.

## Introduction

In this article we present a concept according to which the biologically most damaging hydroxyl radicals generated by free iron, and prevented by magnesium (Mg) ions, may cause neurodegenerative diseases. The primary target of the iron-induced free radicals is blood fibrinogen that becomes converted to fibrin-like material, which interferes with the effective delivery of oxygen by the altered red blood cells (RBCs) and consequently damages the brain’s neurological functions. We also review data on the protective role Mg, particularly in its ionized form. Mg has been shown to inhibit *in vivo* thrombosis (Ravn et al., 1997; Mussoni et al., 2001; Barbagallo et al., 2011). In this paper, the mechanism of its action will be discussed.

Efficient delivery of oxygen to tissues and organs is paramount for normal functioning of living organisms. It is well known that the brain is particularly susceptible to even short periods of hypoxia, and the chronic shortage of oxygen can cause irreversible neurologic consequences (Peers et al., 2007; Grammas et al., 2011). On the other hand it is generally believed that the prolonged ischemia followed by rapid reperfusion causes so-called “oxidative stress” believed to be preventable by the use of antioxidants (Nunomura et al., 2001). Yet potent antioxidants such as vitamin C and E have failed to provide health benefits in the degenerative diseases that are supposedly due to excessive blood oxygenation (Steinhubl, 2008). At the same time numerous natural substances endowed with oxidative properties (e.g., certain polyphenols) have been shown to provide protection against the damage caused by oxygen-centered free radicals (Summers, 2004; Lipinski, 2011). Therefore, in the current paper we present evidence for the neuroprotective effects of certain dietary components, such as Mg, polyphenols, aliphatic, and/or aromatic unsaturated natural substances.

## Vascular and Hemostatic Factors

The prevailing amyloid hypothesis in Alzheimer’s disease (AD) has recently been challenged by several authors (Pimplikar, 2009; Skaper, 2012; Chetelat, 2013). This challenge is supported by the increasing evidence for the role of vascular components (Kovacic and Fuster, 2012; Diomedi and Misaggi, 2013; Sery et al., 2013), as well as hemostatic factors in neurological diseases and particularly in AD (Kalaria, 2003; Gupta et al., 2005; Kling et al., 2013). Thus, isoforms of fibrinogen gamma chain were identified among insoluble proteins in AD brains (Choi et al., 2002), and it was also shown that fibrin interacted with beta-amyloid protein (Merkle et al., 1996). Other researchers have demonstrated the perivascular leakage of fibrinogen around brain microvessels in AD and HIV-related neurological disorders (Fiala et al., 2002), as well as the presence of fibrin in CNS (Inoue et al., 1997). The existence of a protease-resistant complex of fibrinogen and albumin was also found in AD brains (Lipinski and Sajdel-Sulkowska, 2006). Moreover, Paul et al. (2007) documented that fibrin accelerates neurovascular damage in AD. In acute cerebral lesions of multiple sclerosis tissue plasminogen activator (tPA) was co-localized with fibrin(ogen) on large diameter axons, which finding was interpreted as an attempt to remove fibrin deposits and restore normal axon function (Gveric et al., 2001). Brain injury accompanied by cerebrovascular fibrin deposition was revealed in a hypertensive stroke model (Ninomia et al., 2000). Fibrin clearance and/or deposition was suggested to be a key regulatory mechanism for Schwann differentiation and nerve cell damage (Davalos et al., 2012) due to the altered fibrin structure. It is furthermore known that amyloid-beta delays fibrin clot lyses by altering fibrin structure and attenuating plasminogen binding to fibrin (Zamolodchikov and Strickland, 2012). Recently, Hultman et al. (2013) confirmed that fibrin(ogen) is deposited in the AD neurovasculature and interacts with beta-amyloid, resulting in increased formation of blood clots. Also, in postmortem investigations, an increased deposition of fibrin(ogen) was observed in AD cases compared with non-demented controls, and the authors suggested that a strong correlation exists between cerebral amyloid angiopathy severity and fibrin(ogen) deposition. There is therefore extensive evidence of the role of oxidative damage in the brain of patients with AD although the exact process is not fully understood. However, oxidative stress could be linked to alterations of amyloid-beta metabolism and beneficial effects of antioxidants against amyloid-beta toxicity or AD may be of great importance and are shown in many studies (Chakrabarti et al., 2013).

It is also of interest to note that high blood fibrinogen is associated with an increased risk of AD (van Oijen et al., 2005), and that antibodies to beta-amyloid peptide react with individual chains of fibrinogen (Stern et al., 1990). Moreover, amyloid fibrils were shown to contain fibrinogen antigen-reactive material (Ahn et al., 2010; Cortes-Canteli et al., 2010). It is of interest to note that AD is closely associated with atherosclerosis (Kovacic and Fuster, 2012; Yarchoan et al., 2012) in which blood fibrinogen levels are typically increased (Lipinska et al., 1976; van Oijen et al., 2005). Yet some people may develop a “pure” form of AD based strictly on the pathologic consequences of insoluble amyloid-beta without manifestation of the vascular disease as emphasized by Schneider et al. (2007).

Yet, despite intensive research, we still do not understand how fibrin is being formed in cerebral microcirculation, especially following episodes of hypoxia/ischemia. In contrast to coronary circulation, very few blood components are present in the extravascular brain space, as a result of a selective permeability of blood-brain-barrier (BBB). Perhaps it is not surprising that one of such components is tPA, which might serve as the first line of defense against fibrin formed from plasma leaked after damage to BBB (Gveric et al., 2001). On the other hand, it has been suggested that this activator in conjunction with plasmin may aid the clearance of Abeta peptide as shown in the brains of patients with AD (Melcher, 2004). Alternatively, plasmin formed in the cerebral extravascular space can be neurotoxic, by activating metaloproteinases that are known to degrade basement membrane matrix (Kaur et al., 2004).

## Iron and Free Radicals

Several investigators discuss the involvement of two transition metals, iron and copper, in the pathogenesis of atherosclerosis and AD (Brewer, 2007; Barnham and Bush, 2008; Silvestri and Camaschella, 2008; Smith et al., 2010; Weinberg, 2010; Squitti, 2012; Zheng and Monnot, 2012). Out of these two redox metals, iron was shown to be particularly active in a number of degenerative diseases (Kiechl et al., 1997; Brewer, 2007; Ahluwalia et al., 2010; Depalma et al., 2010; Hahalis et al., 2011; Merono et al., 2011) including neurological disorders (Beard and Connor, 2003; Ke and Ming Qian, 2003; Thomas and Jankovic, 2004; Kell, 2010). It should be emphasized that free iron can participate in the formation reactive oxygen species (ROS) that, in turn, initiates so-called *oxidative stress* leading to AD (Casadesus et al., 2004; Castellani et al., 2012), as well as to those associated with aging (Szweda et al., 2002). The concept of labile iron pool was recently developed, which may explain the connection between iron and free-radical generation (Kruszewski, 2003; Breuer et al., 2008; Benarroch, 2009). However, despite the fact that the concept of oxidative stress has been generally accepted, there is no proof of the involvement of oxidation processes in degenerative diseases (Lipinski, 2011). On the other hand, the presence of biomolecules enriched in one or more atoms of oxygen indicates the involvement of a specific type of ROS – the *hydroxyl radical*. Hydroxyl radicals are the most biologically damaging species, particularly with respect to macromolecules such as proteins, nucleic acids, and carbohydrates (Cheeseman et al., 1988; Lipinski, 2011). We have recently documented that trivalent iron ion (Fe^3+^) reacts with the hydroxyl group of water to produce a powerful hydroxyl radical according to the following reaction:

$$Fe^{3+}{+}^{-}\mathrm{OH}+\;\to Fe^{2+}\;{+}^{\cdot }\mathrm{OH}$$

This reaction can be considered as a special case of the Fenton reaction in which hydroxyl radicals are generated from divalent iron ion in the presence of hydrogen peroxide. The need for the oxidizing agent in the Fenton reaction led to a misleading concept that hydroxyl radicals are formed as a, result of the oxidation reaction. Whatever their origin, it should be emphasized that the most important neurological consequence of the action of hydroxyl radicals is their ability to convert soluble fibrinogen into an insoluble fibrin-like polymer (Lipinski and Pretorius, 2012). A characteristic feature of such a polymer is its resistance to the action of proteolytic enzymes that normally degrade fibrin(ogen) into smaller polypeptide fragments.

The damaging effect of hydroxyl radicals can be explained in terms of the following mechanism: the undesirable molecular interactions in blood proteins are prevented by holding their hydrophobic groups inside the interior of protein tridimensional structures stabilized by *intra*-molecular disulfide bonds. Once these bonds are broken, the polypeptide chains become unfolded with the consequent exposure of the hydrophobic domains, which form *inter*-molecular bonds and result in the formation of large a proteolytic enzymes, as is the case with human prion proteins (Das et al., 2010) and bacterial hydrophobins (Kwan et al., 2006). Moreover, in 2001, researchers suggested that free-radical-induced protein aggregates resistant to proteases are responsible for the amyloid formation (Squier, 2001). It is worth noting that one of the risk factors for AD is diabetes mellitus, in which excessive generation of free radicals was may play a major pathogenic role (Lipinski, 2001). This has recently resulted in raising intriguing questions regarding the common denominator in AD and diabetes (Craft, 2012; Adeghate et al., 2013; Vignini et al., 2013).

Another factor has been suggested to be involved in the pathogenesis of AD, which is copper known to generate hydroxyl radicals in hypoxia. This transition metal as well as iron are present in normal brain tissue at normal concentrations, but are significantly increased in cerebrospinal fluid of AD patients (Multhaup, 1995) and in AD brains (Markesbery and Carney, 1999; Altamura and Muckenthaler, 2009). Moreover, transition metals have been implicated in the generation of free-radical and in the aggregation of amyloid protein in the brains of Alzheimer’s patients (Adlard and Bush, 2006). The role of iron in neurotoxicity was recently reviewed (Stankiewicz and Brass, 2009) and the iron overload in the early stages of life was suggested to induce cognitive impairment and the damage of the brain (de Lima et al., 2005). It is possible that under the reducing conditions of hypoxia, these metal ions generate hydroxyl radical with all its pathological consequences (Dajas-Bailador et al., 1998; Ciriolo et al., 2000). An imbalance between free-radical generation and scavenging was suggested to be one of the earliest pathological events in AD (Moreira et al., 2005).

Additional evidence for the involvement of hydroxyl radicals in the pathogenesis of neurodegenerative diseases comes from the experimental investigations showing the protective effect of a free-radical scavenger *ferulic acid* in mouse neuroblastoma 2a cells (Yan et al., 2012). Moreover, evidence for the involvement of free radicals in these diseases comes from experimental studies with the use of *edaravone*, a potent scavenger of hydroxyl radicals (Abe et al., 2004). On the basis of its scavenging properties, this compound was approved in Japan for the treatment of acute cerebral infarcts within 24 h of onset (Watanabe et al., 1996). Diabetic neuropathy was also shown to be prevented by edaravone in an animal experimental study (Saini et al., 2007). Perhaps, the best evidence for the connection between hypoxia and hydroxyl radical production was provided by showing the protective effect of edaravone in fetal lamb brain after umbilical cord occlusion (Nakajima et al., 2006).

## Hemorheologic Disturbances

Although the hydroxyl radical is considered by mainstream scientists as an oxidant, it behaves as a reducing agent with respect to disulfide bridges in plasma proteins leading to the unfolding and scrambled refolding of the polypeptide chains. In this respect this type of free radicals is similar to a dithiol-reducing agent that when added to human whole blood causes rapid aggregation of RBC and an irreversible polymerization of the plasma proteins (Egyud and Lipinski, 1991). Thus, this *in vitro* phenomenon illustrates the pathologic consequences of the exposure of hydrophobic forces that are potentiated by antioxidants and prevented by the *oxidizing* agents (Pretorius et al., 2013a). For such a reason this concept offers an explanation for the failure of antioxidant therapies in the degenerative diseases – including the neurological disorders (Steinhubl, 2008).

At the same time numerous natural products that are not antioxidants were shown to provide protection against cardiovascular and neurological diseases. Particularly, a group of amphiphilic substances, known as “polyphenols” including resveratrol, epigallo-catechin-3-gallate (EGCG), certain anthocyanins, and flavonols has been shown to be effective scavengers of hydroxyl radicals (Lipinski, 2011). In addition, both EGCG and resveratrol were shown to cross BBB, a phenomenon that is not shared by other polyphenols (Mandel et al., 2006; Srividhya et al., 2008; Bieschke et al., 2010). However, most important biological function of polyphenols, is scavenging of hydroxyl radicals by means of aromatic hydroxylation. This reaction may explain the ability of EGCG to disrupt RBC-parafibrin aggregates, as previously reported by us (Pretorius et al., 2013b).

Continuous and unobstructed delivery of oxygen to the brain by the RBCs depends on their membrane fluidity and the state of aggregation that impairs the release of oxygen (Tateishi et al., 2001). Despite decades of intensive research it is not known what exactly causes RBC aggregation and disaggregation. Over 100 years ago Polish physician, Edmund Biernacki, documented that the phenomenon of erythrocyte sedimentation depends on the interaction with fibrinogen (Biernacki, 1897), the level of which in plasma is frequently elevated in degenerative and inflammatory diseases. Years later an argument was presented that it is not just fibrinogen itself but thrombin-induced soluble fibrin monomers that form bridges between individual cells and in this way increase their sedimentation and/or aggregation (Lipinski et al., 1969). The reason for this is that fibrin monomers are more hydrophobic than fibrinogen itself (van Oss, 1990) and thus can more readily interact with hydrophobic patches on RBC membranes. Thus, the dramatic increase of hydrophobicity of soluble parafibrin fibrils generated by hydroxyl radicals from fibrinogen makes them even more potent inducers of RBC aggregation. Whatever the mechanism, it is obvious that fibrinogen plays a critical role in this phenomenon, so the reduction of its reactivity seems to be of great potential in AD and in other neurodegenerative disorders. Davalos et al. (2012) have even suggested eliminating the effect of fibrinogen by the therapeutic defibrination, which is not a very realistic clinical practice.

Increased RBC sedimentation and/or their aggregation have been observed in the degenerative diseases such as atherosclerosis and inflammation, which are known to be associated with AD (Robinson et al., 1995; Andresdottir et al., 2003). Most recently it was shown that thrombosis mediated by RBC is potentiated by ferric chloride (Barr et al., 2013), which confirms our original observation on the role of iron in pathologic blood coagulation (Lipinski and Pretorius, 2012; Pretorius et al., 2013a,b). Other researchers have also emphasized the role of hemorheology in thrombosis and vascular diseases (Erikssen et al., 2000; Natali et al., 2003; Baskurt et al., 2004; Nagy et al., 2010). It is believed that the abnormalities in RBC obstruct oxygen delivery to the brain (Tateishi et al., 2001; Mohanty et al., 2008; Tripathy et al., 2013), which, in turn, causes hypoxia leading to chronic inflammation (Eltzschig and Carmeliet, 2011; Wyss-Coray and Rogers, 2012). Last but not least is the role of blood hemolysis that releases trivalent iron (Woollard et al., 2009) with all its pathologic consequences (Pretorius and Lipinski, 2013). It should be kept in mind that AD, especially when presented during the later stages of life, presents as a co-occurrence with vascular pathology, such as white matter disease. The question that now arises is exactly how damage due to hydroxyl radicals and typical vascular pathology that may develop independently, are interlinked. We suggest that the additional burden of the presence of hydroxyl radical damage might cause a faster progression of the disease.

## Inflammation

A large body of data indicates that inflammation is a hallmark of AD and other neurodegenerative diseases (Tuppo and Arias, 2005; Paul et al., 2007; Ray and Lahiri, 2009; Davalos et al., 2012; Wyss-Coray and Rogers, 2012; Krstic and Knuesel, 2013). In view of a close relationship between inflammation and hypoxia (Eltzschig and Carmeliet, 2011) it is possible that the culprit is a persistent obstruction of cerebral blood flow caused by the chronic formation of iron-induced fibrin/RBC aggregate. In another scenario pathologic fibrin, being resistant to the proteolytic degradation, may present itself to the innate immune system as a foreign body. According to Mechnikov, inflammation is a protective adaptation response to a foreign body that activates macrophages in an attempt to eliminate it from a living organism (Palmblad, 2010). Whereas in most cases the powerful enzymatic machinery of macrophages eventually digests foreign pathogens, the specific hydrophobic properties of iron-induced material make it refractory to the proteolytic degradation, thus initiating a chronic state of inflammation.

## The Protective Role of Magnesium

Mg is a cofactor in numerous enzymes and plays a critical role in many physiologic reactions, including the regulation of cell membrane stability (Woolf, 1961; Elin, 1994; Fawcett et al., 1999). Since Mg is not routinely measured in medical practice, its deficiency may remain undetected for a long period of time (Touyz, 2004; Assadi, 2010). Large epidemiological studies indicate that the inadequate dietary Mg intake and its low serum concentration are associated with insulin resistance, type 2 diabetes mellitus (Corica et al., 2006; Huang et al., 2012) and with cardiovascular diseases (Amighi et al., 2004; Adamopoulos et al., 2009). In addition, hypomagnesemia occurs frequently in association with hypertension (Houston, 2011) and is related to all-cause mortality (Reffelmann et al., 2011). It has also been argued that correcting hypomagnesia may enhance memory (Slutsky et al., 2010) and even prolong life (Rowe, 2012).

Mg deficiency is frequently encountered in critically ill patients (Limaye et al., 2011), particularly those with systemic inflammation (Mazur et al., 2007; Weglicki, 2012). Although Mg deficiency has been known for a long time to be correlated with thrombotic diseases, the mechanism of its hemostatic function is not well understood (Barbagallo et al., 2011). Total plasma Mg concentrations are remarkably constant in healthy subjects and do not substantially change with aging (Romani, 2013) but its ionized form, representing 55% of total Mg, has been shown to be decreased in cardiovascular disease (Kupetsky-Rincon and Uitto, 2012) and in AD as well (Durlach, 1990; Cilliler et al., 2007; Barbagallo et al., 2011).

Health beneficial effects of Mg can be explained by the results of our finding of its anticoagulant properties, as shown in Table 1.

**Table 1:** **Effect of magnesium chloride on spontaneous coagulation of whole blood and on thrombin-clotting times of plasma**.

	Final magnesium chloride concentration (mM)				
	0	2	4	6	8
Coagulation time (s)	250 ± 32	485 ± 47	670 ± 50	1,220 ± 84	>2,000
Clotting time (s)	26 ± 3.5	27 ± 3.5	25 ± 3.6	24 ± 2.7	23 ± 2.4

*To avoid interference of anticoagulants with magnesium the experiments were done with freshly drawn whole blood and dialyzed plasma. Blood was drawn from five healthy subjects (three males and two females, age 47–78) into plain evacuated plastic tubes and immediately placed on ice. Subsequently 0.3 ml portions of whole blood were pipetted into glass test-tubes (10 mm × 70 mm) containing various concentrations of magnesium chloride (Sigma-Aldrich). Afterward the test-tubes were incubated at 37°C and the coagulation times of blood recorded. In a separate experiment human citrated plasma (pooled) was dialyzed against phosphate buffered saline (PBS), pH 7.4, and 0.2 ml portions mixed with 20 μL of magnesium chloride solutions at various millimolar concentrations. Next, 20 μL of thrombin (100 U/ml; Sigma-Aldrich) was added to each tube and clotting time recorded. Each experiment was done in triplicate and mean value ± SD calculated*.

**Figure 1:**
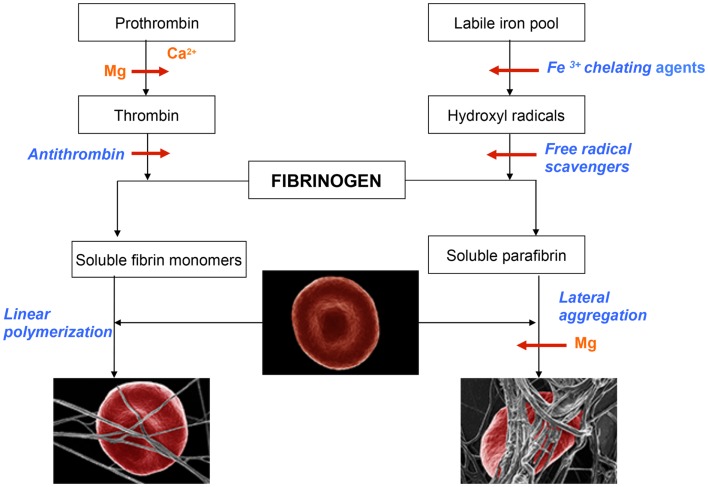
**Schematic representation of events leading to the formation of normal blood clot (left panel) and pathological iron-induced parafibrin (right panel)**. Ca, calcium; Mg, magnesium; RBC, red blood cell.

As shown in Table 1, Mg progressively prolonged coagulation times until the samples became uncoagulable at 8 mM concentration. It is important to note that this novel effect can be observed only with native whole blood and is absent when tested with plasma clotted with thrombin. It has to be concluded, therefore, that the Mg ions interfere with the *intrinsic* activation of prothrombin. This is a very important fact that explains why even large doses of Mg administered to humans have never caused bleeding from the site of vascular injury, at which tissue factor-activated thrombin fulfilled its hemostatic function (Huntsman et al., 1960; Ravn et al., 1997; Ames et al., 1999; Mussoni et al., 2001). The dramatic influence of Mg on blood coagulation may explain its beneficial effects in numerous diseases described by several investigators (Ceremuzynski et al., 2000; Muir et al., 2004; Shechter, 2010; Barbagallo et al., 2011; Kupetsky-Rincon and Uitto, 2012; Muroi et al., 2012; Albrecht et al., 2013). An important fact described by us has to be emphasized – Mg anti-coagulated blood is hemostatically effective as a result of its preserved ability to form functional clots with thrombin that is physiologically generated by tissue factor at the site of vessel wall injury. Apparently, by contrast to coumarin-derived anticoagulants, Mg does not affect prothrombin biosynthesis, but inhibits its conversion to thrombin (Table 1). The role of Mg in hemostasis and in hemorheology is illustrated in Figure 1.

In the normal process of blood coagulation prothrombin is activated to enzyme thrombin that, in turn, converts plasma fibrinogen into soluble fibrin monomer(s). Subsequently, these species undergo spontaneous linear polymerization, which leads to the formation of fibrin fibrils that mechanically trap RBCs (left panel). This phenomenon is very important for effective hemostasis and subsequent fibrinolysis, which is prerequisite for normal wound healing and the return of RBC into the circulation. By contrast to this physiological process, the pathological iron-induced blood coagulation involves polymerization and irreversible entrapment of RBCs (right panel). In this process hydrophobic soluble parafibrin interacts with the hydrophobic epitopes on RBC membranes, forming large aggregates that are resistant to the fibrinolytic degradation, and in this way prevents RBCs from effectively delivering oxygen to the brain.

**Figure 2:**
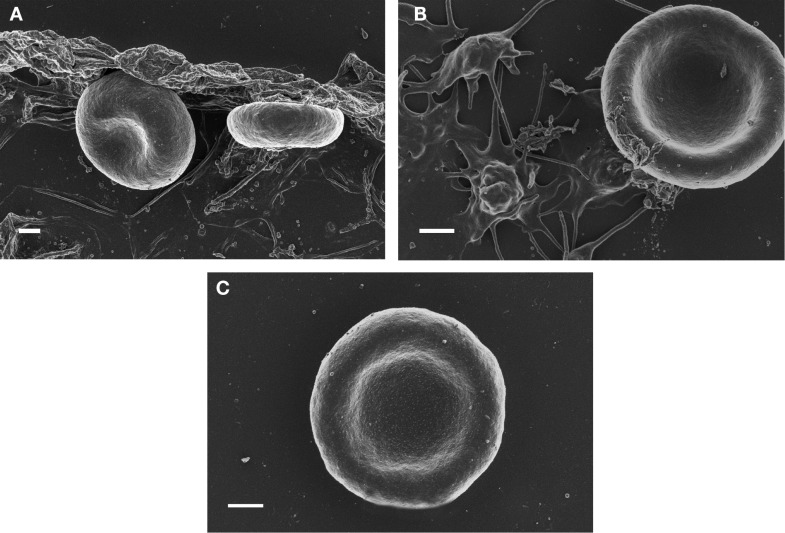
**SEM micrographs of red blood cells from Alzheimer’s disease (A) RBC smear; (B) with 3 mM MgCl_2_; (C) with 30 mM MgCl_2_**. Scale = 1 μm.

Magnesium plays a dual role in hemostasis: first it delays and/or inhibits intravascular generation of fibrin monomers that might have been formed as the results of prothrombin activation; second, Mg ions prevent the interaction of soluble iron-induced parafibrin with RBC. Moreover, and even more importantly, preliminary studies have shown that Mg ions disrupt RBC-parafibrin aggregate as shown in Figure 2 and, in this way, allow erythrocytes to return to the circulation (in whole blood smears of six AD patients and noted in 100 RBCs per individual with and without added Mg). Figure 2 shows representative images for the six patients.

It should be emphasized that a substantial difference exists in the susceptibility to thrombolysis of these two types of clots. In normal blood the intravascular thrombi can be effectively, although only within a specific time window, eliminated with the administration of fibrinolytic therapy. By contrast, abnormal fibrin clots are remarkably resistant to the thrombolytic degradation, remain in the circulation and cause chronic inflammation. This pathologic reaction can be prevented by the chelation of free iron, scavenging of hydroxyl radicals, and/or administration of ionized Mg. Of note, Mg sulfate therapy was shown to be effective in women with eclampsia and pre-eclampsia (Duley et al., 2010; McDonald et al., 2012; Okereke et al., 2012). In addition, low dietary Mg intake was demonstrated to be associated with the risk of stroke (Larsson et al., 2012). Larger doses protect against neurological deficit after brain injury (McIntosh et al., 1989) and were shown to improve insulin sensitivity in non-diabetic subjects (Guerrero-Romero et al., 2004) and in type 2 DM patients (Rodriguez-Moran and Guerrero-Romero, 2003; Kim et al., 2010).

## Other Neuroprotective Factors

It is well known that a healthy diet, rich in polyunsaturated fatty acids (PUFA) and polyphenols may have a positive effect on general health. In particular, the Mediterranean diet has long been known to have a positive effect on the health status of individuals, including that of AD patients (Scarmeas et al., 2006; Thaipisuttikul and Galvin, 2012). Countless papers show that the major components of this diet include mono- and PUFA (in the form of fish oil), minerals such as Mg, polyphenolic substances (red wine) and iron chelating agents contained in citrus fruits (Feart et al., 2010; Gu et al., 2010; Ayissi et al., 2013; Hu et al., 2013). All these substances and agents are known to scavenge hydroxyl radical by virtue of aromatic hydroxylation, as well as to chelate iron (Thaipisuttikul and Galvin, 2012). This specific combination of food products present in the Mediterranean diet results in the inhibition of the generation of parafibrin, which we suggest, contributes to the development and progression of AD. It should also be noted that these beneficial dietary effects may be important in prevention of cardiovascular disease (Lipinski and Pretorius, 2013).

### Polyunsaturated fatty acids

The brain is known to contain elevated quantities of PUFA that paradoxically do not become oxidized by the high oxygen level present in the brain. By contrast, PUFAs have been shown to be neuroprotective, which can be explained in terms of their double bonds acting as scavengers of hydroxyl radicals by converting them to corresponding hydroxy-fatty acids (Czapski, 1984; Yavin et al., 2002; Song et al., 2004). A highly unsaturated eicosapentanoic acid was also shown to offer neuroprotection in the hippocampus of gamma-irradiated rats (Lonergan et al., 2002) and in other experimental models (Marszalek and Lodish, 2005). Concentrations of essential fatty acids in plasma and RBCs and brain tissue were found to be lower in patients with AD (Tully et al., 2003; Issa et al., 2006; Milte et al., 2009) and in the aging brain (Lukiw and Bazan, 2008). It is therefore not surprising that supplementation with fish oil in AD patients substantially improved their memory (Corrigan et al., 1991; Yehuda et al., 1996; Sinn et al., 2010). Moreover, consumption of fish was shown to be associated with a reduced risk of AD (Morris et al., 2003), which might explain the positive effect of the Mediterranean diet on cognitive functions and dementia (Vassallo and Scerri, 2013; Kesse-Guyot et al., 2013; King, 2013; Lourida et al., 2013). There are also other important nutrients in this type of diet, such as minerals (e.g., Mg in the form of chlorophyll contained in green vegetables) (Feart et al., 2012) and polyphenolic substances that can protect the brain against the pathogenic effect of iron overload.

### Polyphenols

Phenolic compounds and/or polyphenols constitute an important group of compounds occurring in plants, comprising at least 8,000 different known structures – including simple phenols, phenolic acids, coumarins and isocoumarins, naphthoquinones, xanthones, stilbenes, flavonoids, and lignins (Rossi et al., 2008; Dudonne et al., 2009). These natural substances exhibit a wide range of biological effects including antibacterial-, anti-inflammatory-, antiallergic-, antifungal-, antithrombotic-, and vasodilatory actions (Lin, 2011; Angeloni et al., 2012; Kumar et al., 2012). More importantly the substances are endowed with a capacity to scavenge hydroxyl radicals (Zielonka et al., 2003; Lipinski, 2011), which might explain their neuroprotective properties (Bieschke et al., 2010; Wang et al., 2012).

Only very few polyphenols can cross BBB, the most active in this respect being EGCG (Mandel et al., 2006; Meng et al., 2010; Palhano et al., 2013; Srividhya and Kalaiselvi, 2013). Other polyphenolic substances, such as curcumin (Baum and Ng, 2004; Ringman et al., 2005; Zhao et al., 2012) have to be first metabolized to small molecular-weight benzoic acid derivatives that are easily absorbable and can enter the brain circulation after crossing BBB (Kahle et al., 2007; Wang et al., 2012). These metabolites are known to scavenge hydroxyl radical (^⋅^OH) by virtue of their addition to double bonds with the formation of a corresponding hydroxyl derivative:

$$R-\mathrm{CH}=\mathrm{CH}-R{+}^{\cdot }\mathrm{OH}\;\to R-\mathrm{CH}-\left(\mathrm{OH}\right)-\mathrm{CH2}-R.$$

It should be emphasized, however, that in the case of polyphenols, only those with available *ortho-*position in their phenolic rings will effectively scavenge hydroxyl radicals. A classic example of such a mechanism, is the aromatic hydroxylation of salicylates this constitutes a principle for the quantitative determination of hydroxyl radicals *in vivo* (Grootveld and Halliwell, 1986; Ueno et al., 2006). Anti-inflammatory effects of aspirin and salicylates (Berk et al., 2013), as well as other small molecular-weight phenolic substances including melatonin (Srinivasan et al., 2005; Galano, 2011), ferulic, chlorogenic, and coumaric acids (Srinivasan et al., 2007), are very likely due to their ability to scavenge hydroxyl radicals by means of aromatic hydroxylation.

## Conclusion

In the present paper we argue that the neurological disturbances in AD can be explained in terms of the hemostatic and hemorheologic effects of free iron in the cerebral circulation. It is known that the iron pool increases with age because no mechanism exists for its elimination from the human body. At a certain critical concentration trivalent iron ions will react with water to give rise to the biologically most reactive hydroxyl radicals that, in turn, unfold and randomly refold fibrinogen polypeptide chains, which results in the formation of a fibrin-like polymer (parafibrin). Such a polymer, by contrast to a thrombin-induced fibril, is remarkably resistant to degradation by the fibrinolytic enzyme system. In addition, soluble parafibrin interacts with hydrophobic patches on RBC membranes, forming huge aggregates that obstruct cerebral blood flow and thus impair oxygen delivery to the brain. In addition, parafibrin deposits residing for longer times in the brain tissue may assume the role of a foreign body, which in turn induces the state of chronic inflammation. Therefore, according to our hypothesis, AD is associated with chronic hypoxia caused by the gradual accumulation of parafibrin that is generated by a non-enzymatic pathway and is totally resistant to the proteolytic degradation. By contrast, fibrin clots formed in the brain by the action of thrombin, as in vascular dementia, are eventually removed by the powerful fibrinolytic enzyme system and may not be found in “pure” AD. We suggest that parafibrin, which is responsible for chronic brain hypoxia, is generated by the mechanisms other that the activation of blood coagulation. Therefore, vascular risk factors are not detectable in patients with pure AD (shown in Figure 1). In order to stop the vicious cycle it is necessary to break this pathologic chain of events by: (1) limiting the dietary supply of trivalent iron; (2) supplying sufficient quantities of natural iron chelating substances (e.g., certain polyphenols); (3) increasing the consumption of omega-3 fatty acids (e.g., fish oil) and other unsaturated aliphatic and/or aromatic substances that will scavenge hydroxyl radicals; and (4) increase the intake of Mg. It should be noted that these conditions are already met in the so-called Mediterranean diet, which is known to be associated with lower incidence of cardiovascular and AD (Lourida et al., 2013). Animal models of AD are frequently used to study the mechanisms underlying AD pathogenesis, genetic interactions with genes of interest, and environmental risk factors that cause sporadic AD, as well as to test the therapeutic effects of AD drug-candidates on neuropathology and cognitive function (Lee and Han, 2013). An important next step with regard to this hypothesis would be to investigate using, for example, animal models, the effect of Mg and iron chelating, and/or hydroxyl radical scavengers.

## Conflict of Interest Statement

The authors declare that the research was conducted in the absence of any commercial or financial relationships that could be construed as a potential conflict of interest.
